# Validation of the Ukrainian Prolonged Grief Assessment for Children (PGA-C) in a Wartime Context

**DOI:** 10.21203/rs.3.rs-10008196/v1

**Published:** 2026-07-20

**Authors:** Oleksandr Avramchuk, R Balahurak, Halyna Shved, Holly Prigerson

**Affiliations:** Ukrainian Catholic UniversityLviv; Ukrainian Catholic UniversityLviv; Ukrainian Catholic UniversityLviv; Weill Cornell Medicine

## Abstract

**Background:**

Prolonged grief reactions in children and adolescents are linked to marked emotional distress, social withdrawal, academic disruption, and impaired daily functioning. These difficulties may be intensified in contexts of chronic threat and instability, such as the ongoing war in Ukraine, where bereavement is widespread and support systems are disrupted. Valid, culturally adapted assessment tools are essential for identifying youth experiencing clinically significant grief responses. This study aimed to adapt and validate the Ukrainian version of the Prolonged Grief Assessment for Children (PGA-C) in a sample of bereaved young people living under wartime conditions.

**Methods:**

A total of 129 bereaved children and adolescents (63.6% female; M age = 12.1, SD = 3.14) completed structured interviews. Analyses included descriptive statistics, internal consistency (Cronbach’s α), test–retest reliability over 4–5 weeks (ICC), exploratory factor analysis (EFA), confirmatory factor analysis (CFA), convergent validity testing, and regression analyses. Convergent/divergent validity was examined through associations with PTSD symptoms (CRIES-8), anxiety and depression (RCADS-25), and health-related quality of life (KIDSCREEN-27).

**Results:**

Internal consistency was excellent for the total PGA-C scale (α = 0.92) and strong for both identified factors (α = 0.89 and 0.88). Test–retest reliability was high (ICC = 0.88, 95% CI [0.82–0.91], p < 0.001). PGA-C scores showed strong negative associations with quality of life (r = −0.622, 95% CI [− 0.729, −0.488]) and positive associations with depression (r = 0.480, 95% CI [0.330–0.620]) and PTSD symptoms (r = 0.438, 95% CI [0.273–0.585]). Correlations of PTSD (r = −0.333, 95% CI [−0.483, −0.162]) and depression (r = −0.350, 95% CI [−0.500, −0.190]) with components of quality of life were clearly weaker than those of PGA-C. No significant differences in PGA-C scores were found for sex, age, relationship to the deceased, or cause of death. EFA of the PGA-C supported a two-factor structure - Detachment/Avoidance and Yearning/Disbelief, yet CFA of the PGA-C showed limited fit of the data to this model (CFI < 0.90, RMSEA > 0.10). Strong internal consistency of the total scale and its concurrent/divergent validity support the use of the PGA-C total score.

**Conclusions:**

The Ukrainian PGA-C demonstrates excellent internal consistency, strong test–retest reliability, and robust convergent and divergent validity. Findings support its use as a reliable tool for assessing prolonged grief among bereaved children and adolescents living in high-adversity wartime contexts.

## Introduction

Armed conflicts have a devastating impact on the mental health of children and adolescents, creating long-term consequences that can affect nearly every domain of development. Over the past 16 years, the United Nations has documented more than 266,000 cases of grave violations against children across more than 30 conflict zones worldwide, including killing, injuring, abduction, sexual violence, and the recruitment and use of children in hostilities [1]. A substantial proportion of the world’s children now live in regions affected by war, violence, or armed conflict, conditions that markedly increase the risk of developing mental disorders [2; 3; 4]. Because children’s understanding of death is still emerging, their grief often manifests differently than adults’, including through aggression, anxiety, somatic symptoms, and social withdrawal [5; 6; 7; 8].

Recent literature reviews on adolescents who have lost parents [9; 10], as well as a synthesis of seven meta-analyses and systematic reviews [11], underscore the profound adversities faced by young mourners in conflict settings. Carpiniello’s systematic review [11] found that among war-exposed children and adolescents, prevalence rates of depression, anxiety, and post-traumatic stress disorder (PTSD) were approximately two to three times higher than in non-exposed populations. In Kosovo, parental death was associated with substantially higher rates of major depressive and anxiety disorders compared to war-exposed but non-bereaved peers (59% vs. 40%) [12]. Findings from Ukraine mirror these patterns: more than 60% of adolescents in the Donetsk region witnessed armed attacks, and nearly 28% lost their homes [13]. Many exhibited a wide range of clinical symptoms, including anxiety and depressive disorders, somatic complaints, enuresis, aggression, apathy, and diminished motivation [14; 15].

Of particular concern is the emergence of severe and persistent grief—now formally recognized as Prolonged Grief Disorder (PGD) in DSM-5-TR and ICD-11—which is associated with substantial distress and functional impairment [16]. Reported PGD prevalence among bereaved adults varies across samples, from nearly 4% in a Yale cohort to 11% in an Oxford sample and 15% in a younger Utrecht cohort [16]. Among children and adolescents, Falala et al.’s 2024 systematic review found PGD prevalence estimates ranging from 10.4% to 32% [17]. Beyond direct exposure to war, children’s mental health is shaped by social, cultural, and interpersonal factors, including displacement, disrupted family dynamics, and loss of social networks [18]. A key limitation in the current literature is that PGD prevalence estimates vary depending on the diagnostic framework used; ICD-11 criteria are more permissive than DSM-5-TR, potentially yielding higher prevalence rates [19]. We here focus on the DSM-5-TR criteria which yield more conservative estimates [19]. In Ukraine, research on prolonged grief in children and adolescents is ongoing, with particular attention to caregiver- and family-reported assessments [20].

Given the ongoing war in Ukraine, rigorous investigation into the mental health consequences for bereaved children and adolescents is urgently needed. Although most children adapt to loss without developing chronic psychopathology, identifiable subgroups are at elevated risk for PGD and may benefit from timely, grief-focused intervention. Analyses of grief manifestations, coping strategies, and risk and protective factors can inform screening practices and guide intervention development. Such work is essential not only for understanding the scope of the problem but also for identifying pathways to mitigate the enduring mental health consequences of war for bereaved young people.

## Methods

### Procedures and Sample

Data were collected in phases between November 2023 and June 2025 in unoccupied territories of Ukraine. Participants were recruited through voluntary requests for psychological support related to bereavement-related emotional distress during the ongoing war. The study was conducted as part of the project “Depths of the Heart: Research into Grief Processes and Mental Health of Children during the War” based in the Department of Clinical Psychology, Ukrainian Catholic University, Lviv, in collaboration with the “International Workgroup on Grief in Children & Adolescents” (Prigerson and Smith, Workgroup Co-Directors). The research protocol complied with the Declaration of Helsinki and received approval from the Institutional Ethics Council of the Ukrainian Catholic University (Protocol №5/23-кп). Informed consent was obtained, and anonymity, confidentiality, and data security were ensured.

A pilot study (the first phase) of the translation and adaptation of the adult and child versions (November 2023–April 2024) of the instruments was first conducted with 40 bereaved children aged 8–18 years (M = 13.4, SD = 2.9; 17 males, 42.5%; 23 females, 57.5%; mean months since loss = 14.9, SD = 6.2) and 60 bereaved adults who were parents or guardians of these children (M = 42.1, SD = 2.1; 22 males, 36.7%; 38 females, 63.3%; mean months since loss = 16.1, SD = 5.15). At retest, data were obtained from 37 children/adolescents and 56 adults.

Participating children completed the Prolonged Grief Assessment – Child Version (PGA-C) [21], the Revised Child Anxiety and Depression Scale – Child Version (RCADS) [22], the Children’s Revised Impact of Event Scale (CRIES-8) [23], and additional KIDSCREEN-27 [24]. Sociodemographic data were obtained from participating children and confirmed from parents or caregivers via a structured questionnaire. Parents and/or caregivers obtained an assessment that contains: the Prolonged Grief Assessment – Adult Version adopted for parents/caregivers, Revised Child Anxiety and Depression Scale – Parent-Version, and KIDSCREEN Health-Related Quality of Life Questionnaire – Parent-Version.

Inclusion criteria: voluntary informed consent; children aged 8–18 years who experienced the death of a significant person to them during wartime (02/24/2022 to present), including loss of both parents; bereavement for at least six months; and informed consent from parents or guardians.

Exclusion criteria: loss outside wartime; ambiguous loss (disappearance without confirmed death); rater determination of intellectual disability or other mental/neurological conditions limiting cognitive ability or questionnaire completion/interviewing.

Most respondents in the pilot study lived in Ukraine (41% West, 19% North, 13.5% South, 11% Central, 3% East in unoccupied territories) or abroad (13.5%). Among children and adolescents, 92% had lost their father, and 8% had lost both parents. This predominance of paternal loss is consistent with findings from the Kosovo study by Morina et al. (2011), which showed that bereaved participants were exposed to significantly more potentially war-related traumatic events than non-bereaved participants (excluding the loss of first-degree relatives) [12]. Living arrangements in the Ukrainian sample included mother only (35%), mother and sibling(s) (30%), or guardians such as grandparents or extended relatives (35%). Mean prolonged grief scores were PGA-C: M = 39.5 (SD = 8.70) and PGA-A: M = 34.8 (SD = 7.20). Test–retest reliability was high: PGA-C ICC = 0.82 (95% CI [0.727–0.90], p < 0.001; α = 0.85) and PGA-A ICC = 0.74 (95% CI [0.62–0.83], p < 0.001; α = 0.81), based on data collected from February 5 to April 16, 2024. These results have been included in a master’s thesis by R. Balahurak (2024, in Ukrainian) and H. Shved (2024, in Ukrainian) deposited in an institutional repository of Ukrainian Catholic University [25; 26].

In the second phase, we recruited 129 children (M ages = 12.1 years, SD = 3.14; 47 males, 36.4%; 82 females, 63.6%; mean months since loss = 14.1, SD = 5.65). Loss type among children: 79.8% father, 8.5% mother, 11.6% both parents; causes included death in combat (77.5%), shelling of civilian areas (18.6%), and accidents (3.9%). All respondents in the second stage lived in Ukraine, mostly in Western regions and Kyiv. The initial interview in the second phase was conducted during the first visit by the psychologist. The second assessment was conducted *prior to commencing a psychological intervention*. Children were reassessed using all instruments according to the protocol. Because of the circumstances of the ongoing war and higher demand for psychological support, an additional interview was conducted after 4–5 weeks after the initial meeting with the sample in 111 participants. We used these data from the PGA-C as a rare opportunity to evaluate test–retest reliability among young people bereaved because of war.

### Measures

The research team of the “International Workgroup on Grief in Children & Adolescents” developed an original sociodemographic questionnaire and assessment battery to collect comprehensive background information about participants. This measure captured key contextual variables relevant to bereavement, persistent trauma-related exposure, and adjustment to war. Data collected included the adolescent’s age, sex, and religion, as well as parental marital status and grieving. To assess grief-specific experiences, participants were asked to report which of their important close others had died, which of these deaths had the greatest impact on them, if both parents died, and whether they attended the funeral. Additional questions addressed prior experiences of losing a relative, physical injuries and mental health problems sustained during wartime, experience of displacement, school absenteeism, and experience of seeking psychological support.

All assessments were administered individually by trained personnel in safe and secure locations. Interviews were conducted either alone with the child/adolescent or in the presence of a parent/guardian, with informed consent. All personnel involved in administering the interviews and assisting participants with questionnaire completion were licensed mental health professionals (i.e., psychologists). As described below, validated, standardized self-report measures were used, and their psychometric properties have been previously established.

### International Workgroup on Grief in Children & Adolescents Assessment Battery included:

#### Prolonged Grief Assessment – Child Version (PGA-C)

The PGA-C is a self-report questionnaire designed for children aged 8–17 years to assess symptoms of prolonged grief. In contexts of younger age or low literacy, interviewer administration was provided to enhance comprehension. The PGA-C aligns with DSM-5-TR diagnostic criteria for Prolonged Grief Disorder (PGD) in children, with items adapted from the well validated adult Prolonged Grief–13 Revised (PG-13-R) for developmental appropriateness [16; 27]. The PGA-C contains 17 items, a combination of dichotomous (yes/no) and 5-point Likert-type responses. Endorsement of at least one Criterion B symptom, three Criterion C symptoms, and Criterion D confirms PGD diagnosis. The validity and reliability of the original version of the scale are ongoing. Preliminary studies suggest good internal consistency and construct validity. Cronbach’s α = 0.85 in our preliminary study (based on data collected from February 5 to April 16, 2024). The scale was translated and adapted into Ukrainian by researchers at the Department of Clinical Psychology, Ukrainian Catholic University. The scale was adapted into Turkish after the earthquake of 2023, and validity and reliability testing are ongoing there as well [28]. Administration in conflict settings was conducted by trained psychosocial staff or mental health professionals to ensure safety and emotional support.

#### Revised Child Anxiety and Depression Scale – Child Version (RCADS)

The RCADS is a 47-item self-report scale for children and adolescents aged 8–18 years, designed to assess symptoms of anxiety and depression across six subscales [22]. Items are scored on a 4-point Likert-type scale (0–3). International studies have demonstrated strong internal consistency (α = 0.78–0.88) and factorial validity across cultural groups.

A shorter RCADS-25 version, which was used in the actual study, has been validated, with 15 anxiety and 10 depression items, showing high internal consistency reliability, α = 0.80–0.86 [29; 30]. The Ukrainian version demonstrated good reliability, ranging from α = 0.84–0.87 [31]. In war contexts, the RCADS can be administered individually or in groups (e.g., school settings).

#### Children’s Revised Impact of Event Scale (CRIES-8)

The CRIES-8 is an 8-item self-report measure for children aged 8–18 years that screens for PTSD risk following trauma [23]. It uses a 4-point scoring system (0–5), with higher scores indicating greater symptom severity. The scale has demonstrated good sensitivity and specificity in disaster and conflict settings. Ukrainian adaptation was conducted by specialists from the Institute of Mental Health of the Ukrainian Catholic University (www.ipz.org.ua) in 2017. The brevity of the CRIES-8 makes it particularly suitable for rapid assessment in war contexts, though administration should occur in supportive environments with referral pathways in place for those at high risk.

#### KIDSCREEN-27 Health-Related Quality of Life Questionnaire – Child Version

The KIDSCREEN-27 is a self-report tool for children and adolescents (8–18 years) designed to assess health-related quality of life across domains of physical, psychological, social, and school functioning [24]. It has been validated in over 30 countries, showing robust reliability and cross-cultural applicability.

Responses are provided on 5-point Likert scales, assessing either frequency (never to always) or intensity (not at all to extremely). One general health item uses a distinct scale (excellent to poor). To ensure consistency, certain items (1, 9, 10, 11) are reverse-coded. To standardize the assessment, raw dimension scores were transformed to a 0–100 scale, where higher scores indicated better health-related quality of life. The Ukrainian version is available on the official site, www.kidscreen.org, though there is a need for testing adaptation in the context of war.

To summarize, the PGA-C, RCADS, CRIES-8, and KIDSCREEN-27 represent validated and complementary measures for assessing grief, depression/anxiety, trauma symptoms, and health-related quality of life in children affected by war. Their administration requires careful attention to literacy levels, emotional safety, and cultural context. The International Consortium for Health Outcomes Measurement (ICHOM) also recommends RCADS, CRIES-8, and KIDSCREEN as standard outcome measures for child and adolescent mental health, further underscoring their clinical and research utility.

### Statistical Methods

All analyses were conducted using Jamovi (version 2.6.26). Descriptive statistics were computed for all variables. Normality was tested using the Shapiro–Wilk test, skewness, and kurtosis; all PGA-C scores were non-normally distributed, supporting non-parametric methods. The factor structure of the PGA-C was examined using exploratory factor analysis (EFA) with principal axis factoring and varimax rotation, guided by parallel analysis, eigenvalues, and scree plots. Confirmatory factor analysis (CFA) was used to test model fit.

Reliability was assessed using Cronbach’s α, item–total correlations, and test–retest intraclass correlation coefficients (ICCs).

Convergent and divergent validity were evaluated via Spearman correlations between PGA-C, RCADS, CRIES-8, and KIDSCREEN-27 scores. To test predictive value, linear regression models were conducted with PGA-C scores as predictors of anxiety, depression, and quality of life outcomes. Group differences were explored using ANCOVA with age as a covariate.

All analyses used two-tailed tests with α = .05.

## Results

Table 1 provides descriptive statistics of the sample. Descriptive statistics revealed a normal distribution of PGA-C total scores as evidenced by the Shapiro–Wilk test (p > 0.05), skewness = − 0.17 and kurtosis = − 0.16 (Appendix A). The distribution of responses across items was generally uniform, with mean scores ranging from 2.29 to 3.49 on a 5-point Likert scale. This pattern suggests that, on average, participants reported mild to moderate symptom severity across PGA-C items. The highest scores were observed for items B1, B2A, C1, C4A, and D1, reflecting intense yearning, sadness, and impairment in daily functioning following loss. Mean prolonged grieving scores in the general sample were PGA-C – M = 43.3 (SD = 11.7): among males – M = 44.0 (SD = 12.4) and females – M = 42.8 (SD = 11.3). Statistical analysis did not reveal significant differences between male and female subsamples.

The suitability for factor analysis was confirmed by the Kaiser–Meyer–Olkin measure (KMO = 0.875) and Bartlett’s test of sphericity (χ^2^ = 1257, df = 105, p < 0.001), indicating adequate intercorrelations among the variables. A Principal Component Analysis (PCA) based on parallel analysis suggested a two-factor model explaining 60.3% of the variance, consistent with the scree plot analysis. In contrast, the Kaiser criterion (eigenvalue > 1) suggested a three-factor model explaining 67% of the variance. The scree plot and parallel analysis results are presented in Appendix A.

Subsequently, an Exploratory Factor Analysis (EFA) was conducted. Given that the normality assumption was not met for all components, the “principal axis factoring” extraction method was used in combination with Varimax rotation. The number of factors was determined based on parallel analysis and corroborated by both the Kaiser criterion (eigenvalue > 1) and the scree plot inspection. The final solution yielded two factors explaining 54.9% of the variance, comprising the same components as in the PCA. The scree plot and parallel analysis for this solution are presented in Appendix A. Factor loadings ranged from 0.50 to 0.81 slightly lower than those obtained in PCA (0.57–0.82). Items B1, C6, C1, C4A, and D1 demonstrated cross-loadings.

Confirmatory Factor Analysis (CFA) showed limited fit of both the unidimensional and the proposed two-factor models. For the unidimensional model, fit indices were χ^2^ (90) = 453, p < 0.001, χ^2^/df = 5.03, CFI = 0.703, TLI = 0.653, SRMR = 0.091, and RMSEA = 0.177. The two-factor model also demonstrated inadequate fit: χ^2^ (89) = 337, p < 0.001, χ^2^/df = 3.78, CFI = 0.798, TLI = 0.761, SRMR = 0.078, and RMSEA = 0.147, all falling below commonly accepted cutoff criteria (Hu & Bentler, 1999; Sivo et al., 2006). Model refinement indicated that item C3 – “Avoidance” had the weakest item–factor loading. Its removal resulted in modest improvements in model fit, reflected in higher CFI and TLI values and a reduction in SRMR; however, overall fit remained at the threshold of acceptability. After removal of item C3, Cronbach’s α for Factor 1 increased slightly to 0.890. Separate CFAs conducted for each factor demonstrated acceptable fit for Factor 1 (χ^2^(20) = 46, p < 0.001, χ^2^/df = 2.30, CFI = 0.95, TLI = 0.93, SRMR = 0.05, RMSEA = 0.10), whereas the model for Factor 2 showed poor fit (χ^2^(20) = 46, p < 0.001, χ^2^/df = 2.30, CFI = 0.70, TLI = 0.65, SRMR = 0.09, RMSEA = 0.18). Given the sample size, these indices should be interpreted with caution. Despite the suboptimal CFA fit, the total scale demonstrated strong internal consistency and validity, as described below.

### Reliability Analysis

The internal consistency of the PGA-C was evaluated using Cronbach’s alpha coefficient, item–total correlations, and the «alpha if item deleted» procedure. The overall Cronbach’s alpha for the 15-item PGA-C was α = 0.92, indicating excellent internal consistency and that all items appear to cohere. All items demonstrated high item–total correlations (> 0.50). Deletion of any item, except the avoidance item (which exhibited the lowest item–rest correlation), resulted in a decrease in the alpha coefficient. Removal of the avoidance item did not substantially alter Cronbach’s alpha. Detailed results, including alpha values. item–total correlations. and the effect of item deletion, are presented in Table 2.

Test–retest reliability was assessed using intraclass correlation coefficient (ICC) analysis on the total scale score, with a 4–5-week interval between administrations in a subsample of n = 111 participants. The analysis yielded an average measures ICC of 0.88 (95% CI: 0.82–0.91), p < 0.001, indicating excellent temporal stability.

The reliability of the two extracted subscales was also examined. Cronbach’s alpha coefficients were α = 0.89 for Factor 1 and α = 0.88 for Factor 2, both reflecting strong internal consistency.

### Convergent and Divergent validity

For the full PGA-C scale, convergent validity was supported by significant positive correlations with measures of posttraumatic symptoms (CRIES-8), anxiety, and depression (RCADS-25 subscales), ranging from r = 0.37 to 0.48 (p < 0.001). Divergent validity was demonstrated through significant negative correlations with physical, psychological, and social well-being domains of the KIDSCREEN-27, ranging from r = − 0.35 to − 0.59 (p < 0.001). Spearman’s rank-order correlations were used as most scale and subscale scores were non-normally distributed based on skewness–kurtosis criteria and the Shapiro–Wilk test (Table 3).

Based on the understanding that the experience of grieving after the loss of a loved one may determine vulnerability to common mental health problems, we conducted a series of regression analyses with the overall prolonged grief score (univariate model) as a predictor. The experience of prolonged grief explained 14% of the variance in the clinical anxiety symptom score (R^2^ = 0.14, F_(1, 127)_ = 20.7, p < 0.001), 22.6% of the variance in clinical depression symptoms (R^2^ = 0.226, F_(1, 127)_ = 37.1, p < 0.001), 37.5% of the variance in physical and 34.7% psycho-emotional well-being (R^2^ = 0.38, F_(1, 127)_ = 76.1, p < 0.001 and R^2^ = 0.35, F_(1, 127)_ = 67.5, p < 0.001). When considering the general regression model for reduced quality of life with predictors associated with the circumstances of the loss experience and living in conditions of persistent trauma-associated distress, the PGA-C indicator demonstrates the largest contribution to the variance (β = − 0.44, p < 0.001) (see Appendix A).

In addition, it was important for us to understand whether these effects were different from emotional distress associated with other traumatic war experiences. A series of regression analyses where the CRIES-8 score was the predictor demonstrated statistically significant patterns of effects (all p < 0.001), but of lower magnitude than PGA-C, for anxiety scores – 10.6%, depression – 15%, and general well-being score – 11%, respectively. To examine divergent validity, we compared the strength of associations between PGA-C scores and quality of life by KIDSCREEN-27 versus posttraumatic stress-related and depressive symptoms. Spearman’s rank-order correlations indicated that the PGA-C total score was significantly negatively correlated with quality of life (r = − 0.53) and positively correlated with depressive symptoms (r = 0.48) and PTSD symptoms (r = 0.44). Depression (r = − 0.29) and PTSD symptoms (r = − 0.26) were also negatively correlated with quality of life; however, these associations were weaker than the correlation between PGA-C and quality of life (see Table 3). To compare dependent correlations, Steiger’s Z-tests with bootstrap 95% confidence intervals (CIs) were conducted for all correlations since we had a non-parametric data distribution.

Results showed a moderate positive correlation between PGA-C and PTSD symptoms (r = 0.44, 95% CI [0.27, 0.59]), indicating that while related, prolonged grief and posttraumatic stress are distinct constructs. PGA-C demonstrated a stronger negative correlation with KIDSCREEN-27 (r = −0.62, 95% CI [−0.73, −0.49]) than PTSD symptoms did (r = −0.33, 95% CI [−0.48, −0.16]), suggesting that PGA-C is more closely associated with diminished quality of life. Same results demonstrated for the depression scale using bootstrap estimates of Spearman correlations and 95% confidence intervals: PGA-C - Depression (RCADS25): r = 0.48, 95% CI [0.33, 0.62]; PGA-C - KIDSCREEN-27: r = −0.62, 95% CI [−0.73, −0.48]; Depression (RCADS25) - KIDSCREEN-27: r = −0.35, 95% CI [−0.50, −0.19]. Because correlation of depression symptoms with quality of life is weaker than grief-related symptoms, highlighting that PGA-C captures unique aspects of functioning that are distinct from self-reported depression. These findings indicate that prolonged grief, as measured by the PGA-C, captures a unique dimension of psychological distress characterized by marked impairment in daily functioning, rather than merely reflecting comorbid depressive or posttraumatic stress symptoms. This pattern supports the divergent validity of the PGA-C and underscores its capacity to assess a construct distinct from related disorders.

Further analyses for the two-factor model PGA-C indicated that Factor 1 (“Detachment/Avoidance”) demonstrated stronger correlations with clinical symptomatology. whereas Factor 2 (“Yearning and Disbelief”) was more closely associated with emotional aspects of well-being. Sequential regression analyses demonstrated that prolonged grief experience was a significant predictor of both anxiety and depression symptoms explaining 15.8% and 24.2% of their variance, respectively (R^2^ = 0.16, F_(2. 126)_ = 11.8, p < 0.001; R^2^ = 0.24, F_(2. 126)_ = 39.5, p < 0.001) in the overall regression models for two factors model. Factor 1 was a significant predictor of anxiety (β = 0.38, p < 0.001, 95% CI [0.16, 0.59]) and depression (β = 0.43, p < 0.001, 95% CI [0.22, 0.63]).

Linear regression models for well-being dimensions revealed that Factor 2 significantly predicted physical well-being (β = − 0.51, p < 0.001, 95% CI [−0.69, − 0.33]), peer relationships (β = − 0.31, p < 0.001, 95% CI [− 0.51, − 0.09]), and school environment well-being (β = − 0.361, p < 0.001, 95% CI [−0.565, − 0.157]). For psychological well-being. both factors were statistically significant (R^2^ = 0.35, F_(2. 126)_ = 33.6, p < 0.001): Factor 1 (β = − 0.31, p < 0.001, 95% CI [−0.50, − 0.12]) and Factor 2 (β = − 0.34, p < 0.001, 95% CI [−0.52, − 0.15]). For autonomy and parent relations. only Factor 1 reached statistical significance (β = − 0.28, p < 0.001, 95% CI [−0.49, − 0.06]).

### Additional Analyses

ANCOVA models with sex as a predictor and age as a covariate revealed no significant effects of sex or age (p > 0.05) on the total PGA-C score. Similarly. no significant differences were observed based on the identity of the deceased person or the cause of death.

## Discussion

The present study examined the psychometric properties of the Ukrainian version of the Prolonged Grief Assessment for Children (PGA-C) in a sample of bereaved children and adolescents during the ongoing armed conflict in Ukraine. Our findings provide strong initial evidence for the reliability and validity of this instrument within a war-affected context. The overall scale exhibited excellent reliability. High test–retest results indicated good temporal stability over a 4–5-week period. Overall, the distribution of the data and their stability are consistent with data from the validation of the adult version of the PG-13-Revised (PG-13-R) scale in three independent samples [16].

Exploratory factor analysis demonstrated a two-factor model, representing “Detachment/Avoidance” and “Yearning and Disbelief” components of the subjective experience of loss — a structure consistent with prior theoretical models of prolonged grief responses [32; 33; 34; 35; 36; 37; 38). Both factors demonstrated satisfactory internal consistency, with Cronbach’s alpha values exceeding the recommended threshold of 0.80 [39]. However, confirmatory factor analysis (CFA) fit indices did not meet conventional criteria and should therefore be interpreted with caution [40]. The removal of item C3 - Avoidance, only modestly improved model fit, suggesting that further refinement and potential cultural adaptation of specific items may enhance structural validity. These results are consistent with results from the San Diego Widowhood Study which identified avoidance as the least informative item examined in the set, and setting the construct apart from PTSD [41].

The pattern of correlations between the PGA-C and external measures supported its convergent validity. As hypothesized, higher grief severity was positively associated with PTSD symptoms, anxiety, and depression, and negatively related to multiple aspects of health-related quality of life. The magnitude of these associations aligns with previous research linking prolonged grief to elevated emotional distress and functional impairment [42; 43; 44].

To further establish divergent validity, we compared the strength of associations between prolonged grief, PTSD, depression, and quality of life. This pattern aligns with previous evidence suggesting that prolonged grief in children and adolescents is not merely a manifestation of PTSD or depression, but rather a unique construct associated with pervasive impairments in social and functional domains [45]. Our findings reinforce this distinction by demonstrating that grief-related symptoms explained greater variance in children’s well-being than either PTSD or depression. Thus, the PGA-C appears to assess a qualitatively distinct dimension of emotional distress, one that reflects the enduring disruption of daily functioning associated with the yearning, disbelief, and avoidance of reminders of the deceased person that characterize prolonged grief, rather than simply capturing general emotional distress or trauma-related symptomatology. Notably correlations in the pilot study were stronger than in the main sample, possibly reflecting differences in the temporal and social context, the transition from the acute phase of war to a prolonged state of conflict. This suggests that environmental stressors beyond bereavement may modulate the intensity of grief-related distress.

Regression analyses highlighted distinct functional roles of the two grief dimensions. Detachment/Avoidance (Factor 1) emerged as a stronger predictor of clinical symptomatology (anxiety and depression), while Yearning and Disbelief (Factor 2) was more closely related to psychosocial aspects of well-being, including physical health, peer relationships, and school engagement. This differentiation may be clinically meaningful as it suggests that interventions targeting avoidance-related behaviors might primarily mitigate internalizing symptoms, whereas addressing yearning and disbelief may be more effective in improving broader social and functional outcomes [46;47;48].

Our finding of no significant differences in grief severity by sex, age, role of the deceased, or cause of death contrasts with some previous studies reporting higher grief and PTSD symptoms among adolescents [49; 50]. In line with prior research, children’s grief reactions are shaped by multiple contextual factors such as the circumstances of the death, relationship to the deceased, caregiving environment, and broader cultural context [37; 51; 52]. This may reflect the overriding influence of war, where persistent environmental stressors and disrupted support systems diminish the relative impact of individual and relational differences. Consistent with prior work, the loss of primary attachment figures remains a critical risk factor; however, contextual factors such as disrupted caregiving, chronic environmental stress, and limited social support may amplify grief severity independently of age, sex, or prior bereavement history, especially in emergency settings [28]. These findings suggest that, under conditions of ongoing armed conflict, contextual moderators traditionally emphasized in grief research may operate differently, highlighting the need for further cross-cultural and longitudinal investigations among children exposed to war.

Similarly, longitudinal analyses among bereaved adults have suggested that trajectories of grief may differ by sex, with men initially showing higher severity but women maintaining or even increasing symptomatology over time [53]. In contrast, other studies using latent class approaches in bereaved children found that symptom profiles (e.g., prolonged grief, PGD+PTSD comorbidity) were not significantly associated with demographic factors, but rather with contextual stress and coping resources [44].

One explanation of these discrepancies is that in war-affected contexts, the overwhelming presence of chronic threat and instability may overshadow demographic or loss-related predictors of grief severity. Thus, our results may reflect the salience of contextual stressors in shaping bereavement responses, pointing to the need for context-specific adaptations of grief assessment tools, as well as interventions, in conflict zones.

### Limitations and Future Directions

Several limitations should be acknowledged. First, although the sample size was adequate for factor analysis, replication in larger and more diverse populations is necessary to confirm the stability of the factor structure. In addition to this limitation, it is worth including that the sample was not randomized and included all participants who provided informed consent when seeking psychological help, which likely inflated the prevalence of PGD. The follow-up assessment for test-retest reliability was after receipt of the initial psychological consultation session. Nevertheless, psychoeducation and general recommendations provided during the initial psychological consultation may have constituted a minimal form of intervention. Although the impact of such support was not assessed, it may have influenced participants’ symptom trajectories over time. If effective, such support would be expected to reduce symptom severity and thus attenuate, rather than inflate, estimates of temporal stability. Therefore, the observed stability of PGD symptoms should be interpreted in light of this circumstance. Third, the influence of war-related stressors may limit the generalizability of these findings to bereaved children in non-war settings. Fourth, it is important to note the limitations and potential biases in interpreting the results, as research on bereavement among children and adolescents is under-researched. Finally, although our findings support the convergent validity of the PGA-C, further examination of discriminant validity and sensitivity to change is warranted.

Future research should explore longitudinal trajectories of prolonged grief in youth, particularly in contexts of persistent stress responses, and how interventions modify symptom severity of PGD. Refinement of the instrument’s factor structure, potentially through item modification or removal, may enhance its cultural and contextual applicability.

## Conclusion

The Ukrainian version of the PGA-C demonstrates strong psychometric properties and appears to be a reliable and valid measure of prolonged grief in bereaved children and adolescents, even within the unique stress context of ongoing armed conflict. The instrument’s two-factor structure reflects core dimensions of prolonged grief, with distinct associations to clinical and functional domains, highlighting its potential utility for both research and clinical practice. Continued evaluation and refinement are recommended to optimize its application across diverse settings.

## Supplementary Material

This is a list of supplementary files associated with this preprint. Click to download.

- AppendixATables.docx

## Figures and Tables

**Table 1:** Characteristics of the Ukrainian sample of bereaved children and adolescents (n = 129)

Age	n	%
	
Primary school age: 8–10 years	48	37.21
Middle school: 11–15 years	55	42.64
High school: 16–18 years	26	20.15
**Sex**		
Female	82	63.6
Male	47	36.4
**“Who died?”**		
One parent:		
Father	103	79.84
Mother	11	8.53
Both parents	15	11.63
**Months after the death**		
6 months to a year	58	44.96
1 to 2 years	63	48.83
more than 2 years	8	6.20
**Causes of death**		
Death in combat	100	77.50
Shelling of civilian areas	24	18.60
Accidents	5	3.90
**“Who does the child currently live with?”**		
One parent	91	70.54
Without parents, with older siblings	19	14.72
Without parents, with grandparents	14	10.85
Without parents, with other relatives (uncle/aunt, etc.)	5	3.88

**Table 2:** Internal consistency of the PGA-C subscales scores in Ukrainian sample of bereaved children and adolescents

Items PGA-C		Mean	SD	Item-rest correlation	If item dropped Cronbach’s α	Shapiro-Wilk, p
**Yearning**	**B1**	3.49	1.13	0.76	0.92	< .001
**Preoccupation - deceased**	**B2A**	3.48	1.11	0.59	0.92	< .001
**Preoccupation - manner of death**	**B3A**	2.93	1.16	0.65	0.92	< .001
**Preoccupation - manner of death**	**B3B**	2.57	0.99	0.60	0.92	< .001
**Identity Disruption**	**C1**	3.12	1.15	0.66	0.92	< .001
**Disbelief**	**C2**	3.01	1.39	0.60	0.92	< .001
**Avoidance**	**C3**	2.29	1.14	0.47	0.92	< .001
**Emotional pain**	**C4A**	3.18	0.83	0.59	0.92	< .001
**Emotional pain**	**C4B**	3.05	1.04	0.61	0.92	< .001
**Difficulty moving on**	**C5A**	2.47	1.03	0.65	0.92	< .001
**Difficulty moving on**	**C5B**	2.70	1.29	0.67	0.92	< .001
**Emotional numbness**	**C6**	2.34	1.16	0.67	0.92	< .001
**Meaninglessness**	**C7**	2.71	1.06	0.72	0.92	< .001
**Loneliness**	**C8**	2.72	1.12	0.62	0.92	< .001
**Impairment**	**D1**	3.22	1.10	0.81	0.91	< .001

**Table 3:** Correlations of the PGA-C total scores in Ukrainian sample of bereaved children and adolescents

		15 items PGA-С	CRIES-8	RCADS25 anxiety	RCADS25 depression
**CRIES-8**	Spearman’s rho	0.44	-		
	p-value	< .001	-		
**RCADS25 – anxiety**	Spearman’s rho	0.37	0.30	-	
	p-value	< .001	< .001	-	
**RCADS25 - depression**	Spearman’s rho	0.48	0.387	0.77	-
	p-value	< .001	< .001	< .001	-
**KIDSCREEN-27**	Spearman’s rho	−0.49	−0.259	−0.22	−0.29
	p-value	< .001	0.003	0.01	< .001
**KIDSCREEN-27 - Physical well-being**	Spearman’s rho	−0.55	−0.308	−0.23	−0.27
	p-value	< .001	< .001	< 0.01	< .001
**KIDSCREEN-27 - Psychological well-being**	Spearman’s rho	−0.59	−0.417	−0.37	−0.47
	p-value	< .001	< .001	< .001	< .001
**KIDSCREEN-27 - Autonomy and parent relations**	Spearman’s rho	−0.35	−0.151	−0.23	−0.28
	p-value	0.01	0.09	< .001	0.001
**KIDSCREEN-27 - Peers and social support**	Spearman’s rho	−0.44	−0.181	−0.18	−0.19
	p-value	< .001	0.04	0.04	0.04
**KIDSCREEN-27 - School environment**	Spearman’s rho	−0.42	−0.257	−0.13	−0.17
	p-value	< .001	0.003	0.15	0.06

## Data Availability

The datasets generated or analyzed during this study are included in this published article. Other data used and/or analyzed during the current study are available from the corresponding author on reasonable request.

## Abbreviations

PGD: Prolonged Grief Disorder
PTSD: Post-traumatic stress disorder
DSM-5-TR: Diagnostic and statistical manual of mental disorders, Fifth Edition, Text Revision
ICD-11: International Classification of Diseases, Eleventh Revision
PG-13-R: Prolonged Grief-13-Revised scale
PGA-C: Prolonged Grief Assessment – Child Version (Nader & Prigerson, 2020)
RCADS: The Revised Child Anxiety and Depression Scale – Child Version (22)
CRIES-8: The Children’s Revised Impact of Event Scale (23)
ANCOVA: Analysis of covariance
ICC: Intraclass correlation coefficient
KMO: Kaiser–Meyer–Olkin measure
EFA: Exploratory Factor Analysis
CFA: Confirmatory factor analysis
PCA: Principal Components Analysis
